# AKAP12, mediated by transcription factor 21, inhibits cell proliferation, metastasis, and glycolysis in lung squamous cell carcinoma

**DOI:** 10.1515/biol-2022-0912

**Published:** 2025-04-09

**Authors:** Juan Chen, Hehe Liao, Kaibin Wang, Tan Yan, Shaofei Ma, Guodong Bai

**Affiliations:** Department of Respiratory Medicine, No. 215 Hospital of Shaanxi Nuclear Industry, Xianyang, Shaanxi, 712000, China; Department of Oncology, No. 215 Hospital of Shaanxi Nuclear Industry, Xianyang, 712000, Shaanxi, China

**Keywords:** lung squamous cell carcinoma, AKAP12, TCF21, growth

## Abstract

A-kinase anchor protein 12 (AKAP12) has been reported to be related to lung squamous cell carcinoma (LUSC) progression. However, its role and molecular mechanisms in LUSC have not been revealed. The mRNA and protein levels of AKAP12 and transcription factor 21 (TCF21) were tested by quantitative real-time PCR and western blot. Cell counting kit 8 assay, EdU assay, flow cytometry, wound healing assay, and transwell assay were used to evaluate cell proliferation, apoptosis, migration, and invasion. Cell glycolysis was measured by testing glucose consumption and lactate production. The interaction between AKAP12 and TCF21 was assessed by ChIP assay and dual-luciferase reporter assay. A mice xenograft model was constructed to explore AKAP12 and TCF21 roles *in vivo*. Our data showed that AKAP12 was underexpressed in LUSC tissues and cells, and its overexpression inhibited LUSC cell growth, metastasis, and glycolysis. TCF21 had decreased expression in LUSC, which facilitated AKAP12 expression through binding to its promoter region to enhance its transcription. Furthermore, TCF21 increased AKAP12 expression to repress LUSC cell growth, metastasis, and glycolysis. *In vivo* experiments showed that AKAP12 upregulation reduced LUSC tumorigenesis, and TCF21 knockdown reversed this effect. In conclusion, AKAP12 might be a tumor suppressor in LUSC, which was mediated by TCF21 and could inhibit cell growth, metastasis, and glycolysis to restrain LUSC malignant progression.

## Graphical abstract

**Figure:**
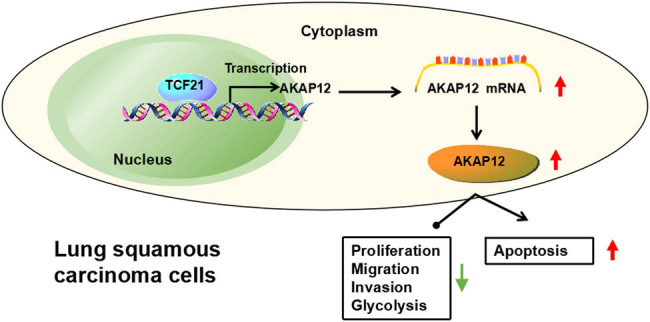

## 1 Introduction

According to the degree of differentiation and biological characteristics, lung cancer is currently divided into two categories, namely small-cell lung cancer (SCLC) and non-small-cell lung cancer (NSCLC) [1]. Lung squamous cell carcinoma (LUSC) is the second most prevalent type of lung cancer and is a pathological subtype of NSCLC [2,3]. Most LUSC patients are diagnosed at an advanced stage, resulting in high mortality [4,5]. At present, patients with advanced LUSC have no chance of radical surgery, and the treatment options are very limited due to lacking effective targeted drugs [6]. Gefitinib and erlotinib targeting epidermal growth factor receptors have been used in the treatment of lung cancer, and EGFR polymorphism has been found to be associated with toxicity associated with tyrosine kinase inhibitors [7]. Circulating neuroendocrine markers chromogranin A (CGA), pro-gastrin-releasing peptide, and neuron-specific enolase may play a potential role in the stage and prognosis of SCLC patients [8]. Although immunotherapy has brought new hope to advanced LUSC, it is not available to all patients [9]. Therefore, the search for specific molecular targets is of great importance for LUSC treatment.

A-kinase anchor protein 12 (AKAP12) is a novel potent scaffold protein for key signaling factors [10]. Studies had found that AKAP12 was lowly expressed in many cancers and had anti-tumor activity, such as esophageal squamous carcinoma cell [11] and breast cancer [10]. Importantly, AKAP12 was confirmed to be downregulated in lung adenocarcinoma, and its inhibition might block cancer malignant progression [12]. Besides, the methylation of AKAP12 was associated with the prognosis of lung cancer patients [13]. Chen et al. found that AKAP12, a pyroptosis-related gene, could be used as a prognostic marker for LUSC [14]. Through database analysis, we found that AKAP12 was underexpressed in LUSC tissues. However, its role and mechanism in LUSC remain unclear.

Transcription factor 21 (TCF21) is a member of the basic helix-loop-helix protein family [15], which is expressed in many tissues and may be involved in regulating lineage-specific gene expression by forming heterodimers with proteins [16]. TCF21 has been found to mediate many biological processes by serving as a tumor suppressor [17]. TCF21 protein expression was confirmed to be reduced in lung cancer cells [18]. Through the LinkedOmics database, we discovered that there was a positive correlation between TCF21 and AKAP12 expression in LUSC tissues. However, as a transcription factor, whether TCF21-regulated LUSC progression by mediating AKAP12 transcription and expression is unknown.

Here, we revealed the role and mechanism of AKAP12 in LUSC progression. Based on the above, we hypothesized that AKAP12 might be regulated by TCF21 to inhibit the malignant progression of LUSC. Our study hopes to provide new ideas for developing clinical treatment options for LUSC.

## 2 Materials and methods

### 2.1 Samples

LUSC patients (*n* = 54) were recruited from No. 215 Hospital of Shaanxi Nuclear Industry, and their tumor tissues and adjacent normal tissues were obtained after surgical resections and stored at −80°C.


**Informed consent:** Informed consent has been obtained from all individuals included in this study.
**Ethical approval:** The research related to human use has been complied with all the relevant national regulations, institutional policies and in accordance with the tenets of the Helsinki Declaration, and has been approved by the Ethics Committee of No. 215 Hospital of Shaanxi Nuclear Industry.

### 2.2 Cell culture and transfection

Human normal bronchial epithelial cells (BEAS-2B) and LUSC cells (H1703 and H520) were purchased from ATCC (Manassas, VA, USA). BEAS-2B cells were cultured in BEGM medium (Lonza, Basel, Switzerland), and LUSC cells were grown at RPMI-1640 medium with 10% FBS (Gibco, Carlsbad, CA, USA). For *in vitro* experiments, transfections of AKAP12/TCF21 overexpression vector and shRNA were performed using Lipofectamine 3000 (Invitrogen, Carlsbad, CA, USA).

### 2.3 Quantitative real-time PCR (qRT-PCR)

Total RNA was extracted by TRIzol reagent (Invitrogen). The extracted RNA was reverse-transcribed with the RevertAid RT Kit (Thermo Fisher Scientific, Waltham, MA, USA). SYBR Green (Thermo Fisher Scientific) was used for qRT-PCR with specific primers (Table 1). Relative expression was tested by the 2^−ΔΔCt^ method. At present, various methods are used in the examination of lung tumors, including qRT-PCR [19].

**Table 1:** Primer sequences used for qRT-PCR

Name		Primers for PCR (5′–3′)
AKAP12	Forward	GAGATGGCTACTAAGTCAGCGG
	Reverse	CAGTGGGTTGTGTTAGCTCTTC
TCF21	Forward	TCCTGGCTAACGACAAATACGA
	Reverse	TTTCCCGGCCACCATAAAGG
β-Actin	Forward	CTTCGCGGGCGACGAT
	Reverse	CCACATAGGAATCCTTCTGACC

### 2.4 Western blot (WB)

H1703 and H520 cells were lysed with RIPA (Beyotime, Shanghai, China) to extract proteins. Extracted proteins were separated and transferred to PVDF membranes. The membrane was blocked and incubated with antibodies. After that, the ECL reagent (Beyotime) was used to detect protein signals. Antibodies were listed as follows: anti-AKAP12 (ab198895, 1:600, Abcam), anti-TCF21 (ab182134, 1:2000, Abcam), anti-β-actin (66009-1-Ig, 1:20,000, Proteintech, Rosemont, IL, USA), and secondary antibody (ab205718 or ab205719, Abcam). The method of WB was consistent with previously described [20].

### 2.5 Cell counting kit 8 (CCK8) assay

H1703 and H520 cells in 96-well plates were treated with CCK8 reagent (Beyotime) after incubation for 24, 48, 72, and 96 h, respectively. Cell viability was evaluated by detecting the OD value at 450 nm by a microplate reader.

### 2.6 EdU assay

H1703 and H520 cells in 96-well plates were labeled with EdU solution and DAPI solution using EdU Cell Proliferation Kit (Beyotime). A fluorescence microscope was used to observe the fluorescence signals, and the EdU positive cell rate was analyzed by ImageJ software.

### 2.7 Flow cytometry

H1703 and H520 cells (5 × 10^5^ cells) were collected and resuspended with binding buffer (Beyotime). Cells were incubated with Annexin V-FITC (Beyotime) away from light for 15 min. Afterwards, cells were treated with PI dyeing solution (Beyotime). Cell apoptotic rate was analyzed by FACScalibur flow cytometer (BD Biosciences, San Diego, CA, USA) with CellQuest Pro software (BD Biosciences). The method of flow cytometry was consistent with previously described [21].

### 2.8 Wound healing assay

H1703 and H520 cells were inoculated in 24-well plates. Using the ruler as a reference, a wound was created using a pipette. After being cultured for 24 h, the cell wound area was photographed to count the wound closure rate.

### 2.9 Transwell assay

H1703 and H520 cells were inoculated into the upper of transwell chamber (BD Biosciences, San Jose, CA, USA) pre-coated with diluted Matrigel, and the completed medium was filled into the lower chamber. After being cultured for 24 h, invaded cell numbers were counted under a microscope.

### 2.10 Cell glycolysis detection

According to the product instructions, Glucose Content Assay Kit and Lactic Acid Content Assay Kit (Solarbio, Beijing, China) were used to evaluate glucose consumption and lactate production in H1703 and H520 cells, respectively.

### 2.11 ChIP assay

Based on ChIP Kit (Beyotime) instructions, H1703 and H520 cells were incubated with formaldehyde, and the cross-linked chromatins were fragments. Supernatants were extracted and incubated with anti-TCF21, anti-IgG, and protein A + G agarose. The precipitate was eluted to collect the chromatin-containing supernatant. Then, the enrichment of the AKAP12 promoter was examined by qRT-PCR.

### 2.12 Dual-luciferase reporter assay

The promoter sequence of AKAP12 bound to TCF21 was cloned into a pGL3-basic vector (Promega, Madison, WI, USA) to construct a WT/MUT-AKAP12 vector. 293T cells were co-transfected with sh-NC/sh-TCF21/Mock/TCF21 and WT/MUT-AKAP12. Luciferase activity was tested using the related kit (Promega).

### 2.13 Mice xenograft models

BALB/c nude mice (4 weeks; Vital River, Beijing, China) were subcutaneously injected with H1703 cells transfected with lentivirus AKAP12 overexpression vector and sh-TCF21 in the right side of the back (*n* = 5/group). Tumor volume and weight were evaluated every 7 days. After 35 days, mice were sacrificed to collect tumor tissues. Additionally, tumor tissues were prepared as paraffin sections to perform immunohistochemical (IHC) staining with anti-TCF21 (ab182134, Abcam), anti-AKAP12 (ab198895, Abcam), anti-Ki67 (27309-1-AP, Proteintech), and anti-E-cadherin (20874-1-AP, Proteintech).


**Ethical approval:** The research related to animal use has been complied with all the relevant national regulations and institutional policies for the care and use of animals. Animal studies were approved by the Animal Ethics Committee of No. 215 Hospital of Shaanxi Nuclear Industry and performed according to the Guide for the Care and Use of Laboratory Animals.

### 2.14 Statistical analysis

Data were expressed as mean ± SD in GraphPad Prism 7 software. Statistical analyses were performed using Student’s *t*-test or analysis of variance. Statistical significance was set as *P* < 0.05.

## 3 Results

### 3.1 AKAP12 had decreased expression in LUSC tissues and cells

**Figure 1:**
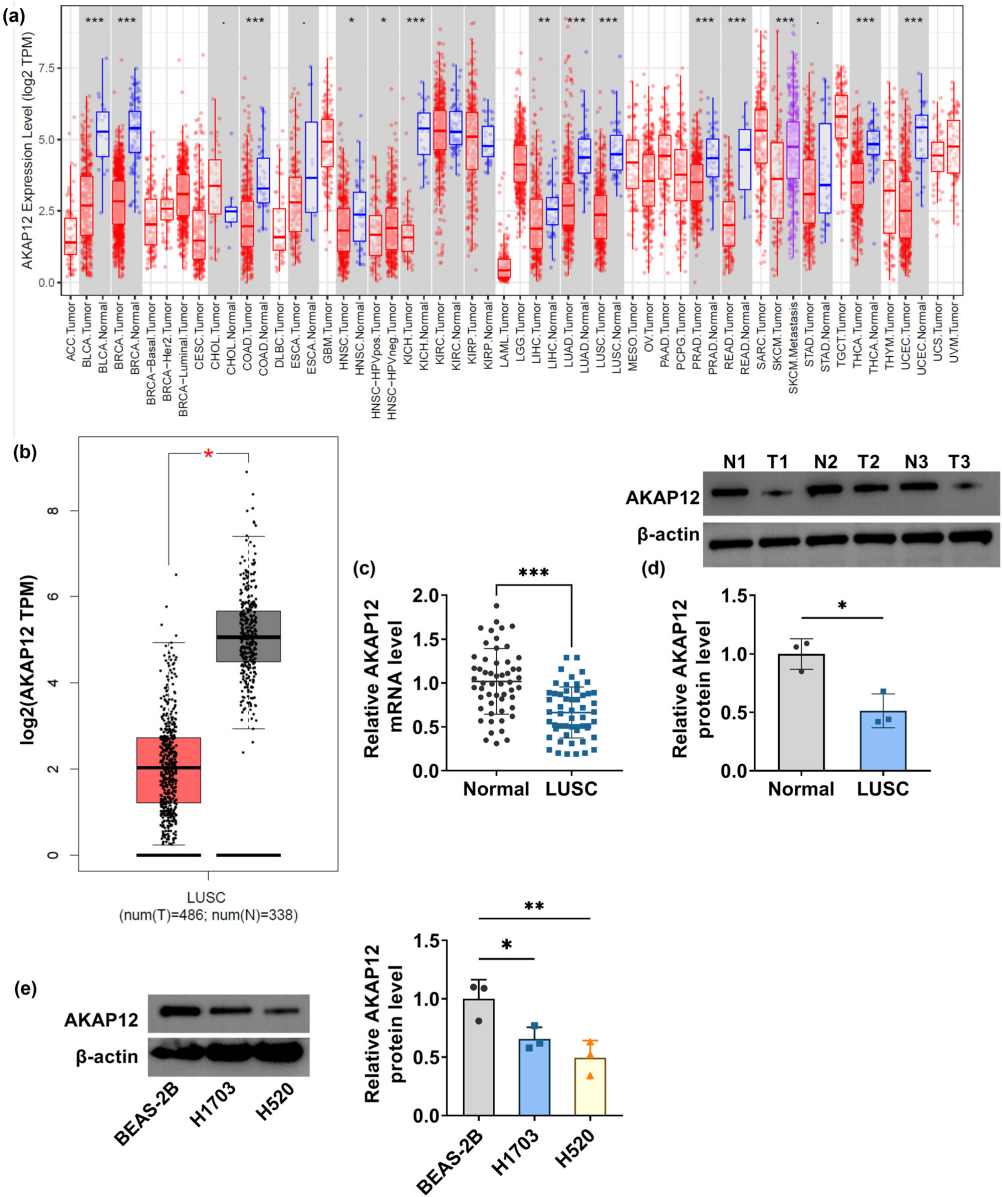
AKAP12 expression in LUSC tissues and cells. (a) TIMER database showed AKAP12 expression in TCGA pan-cancer dataset. (b) GEPIA database revealed AKAP12 expression in LUSC tumor tissues and normal tissues. (c) AKAP12 mRNA level in LUSC tumor tissues (*n* = 54) and adjacent normal tissues (*n* = 54) was detected by qRT-PCR. (d) AKAP12 protein level was detected by WB in LUSC tumor tissues (*n* = 3) and adjacent normal tissues (*n* = 3). (e) AKAP12 protein level in BEAS-2B, H1703, and H520 cells was measured using WB. *P < 0.05, **P < 0.01, ***P < 0.001.

The TIMER database showed that AKAP12 was differentially expressed in the Cancer Genome Atlas (TCGA) pan-cancer dataset (Figure 1a). Besides, the GEPIA database revealed that AKAP12 was lowly expressed in LUSC tumor tissues compared to normal tissues (Figure 1b). Through qRT-PCR and WB, we detected a downregulated mRNA and protein expression of AKAP12 in LUSC tissues (Figure 1c and d). Moreover, AKAP12 protein expression was lower in LUSC cells (H1703 and H520) than in BEAS-2B cells (Figure 1e).

### 3.2 AKAP12 suppressed LUSC cell growth, metastasis, and glycolysis

**Figure 2:**
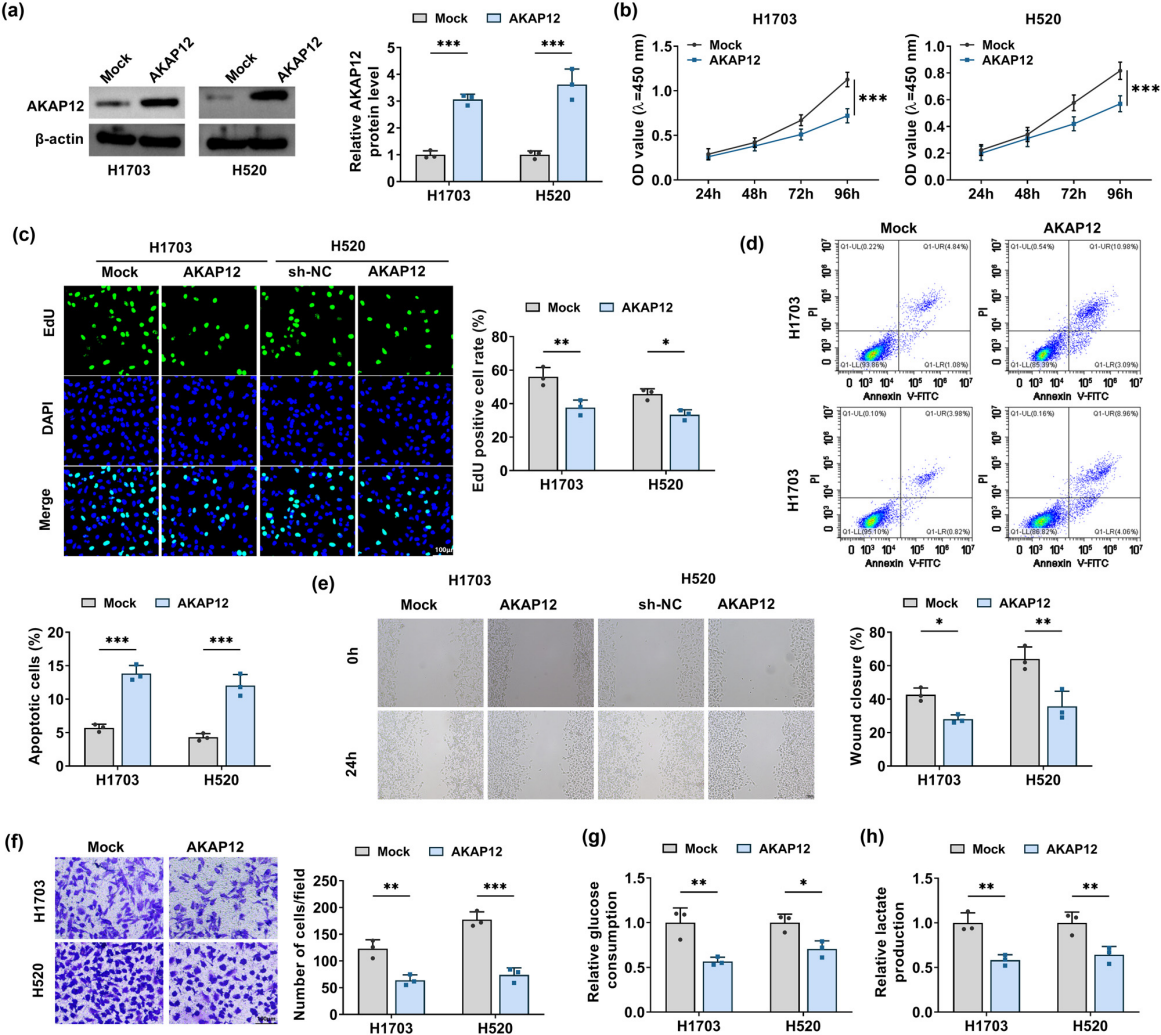
Effects of AKAP12 on LUSC cell growth, metastasis, and glycolysis. H1703 and H520 cells were transfected with Mock and AKAP12 overexpression vectors. (a) AKAP12 protein level was detected by WB. CCK8 assay (b), EdU assay (c), flow cytometry (d), wound healing assay (e), and transwell assay (f) were used to measure cell proliferation, apoptosis, migration, and invasion. (g and h) Cell glycolysis was assessed via testing glucose consumption and lactate production. *P < 0.05, **P < 0.01, ***P < 0.001.

Further analysis was performed to reveal AKAP12 roles in LUSC cell behaviors. We overexpressed AKAP12 at the protein level in H1703 and H520 cells by transfection of AKAP12 overexpression vector (Figure 2a). The results of CCK8 assay, EdU assay, and flow cytometry suggested that AKAP12 overexpression decreased LUSC cell viability, reduced EdU positive cell rate, and enhanced apoptotic cell rate (Figure 2b–d). Meanwhile, we also observed that upregulation of AKAP12 inhibited wound closure rate, invaded cell numbers, glucose consumption, and lactate production in H1703 and H520 cells (Figure 2e–h).

### 3.3 TCF21 was positively correlated with AKAP12 expression in LUSC tissues

**Figure 3:**
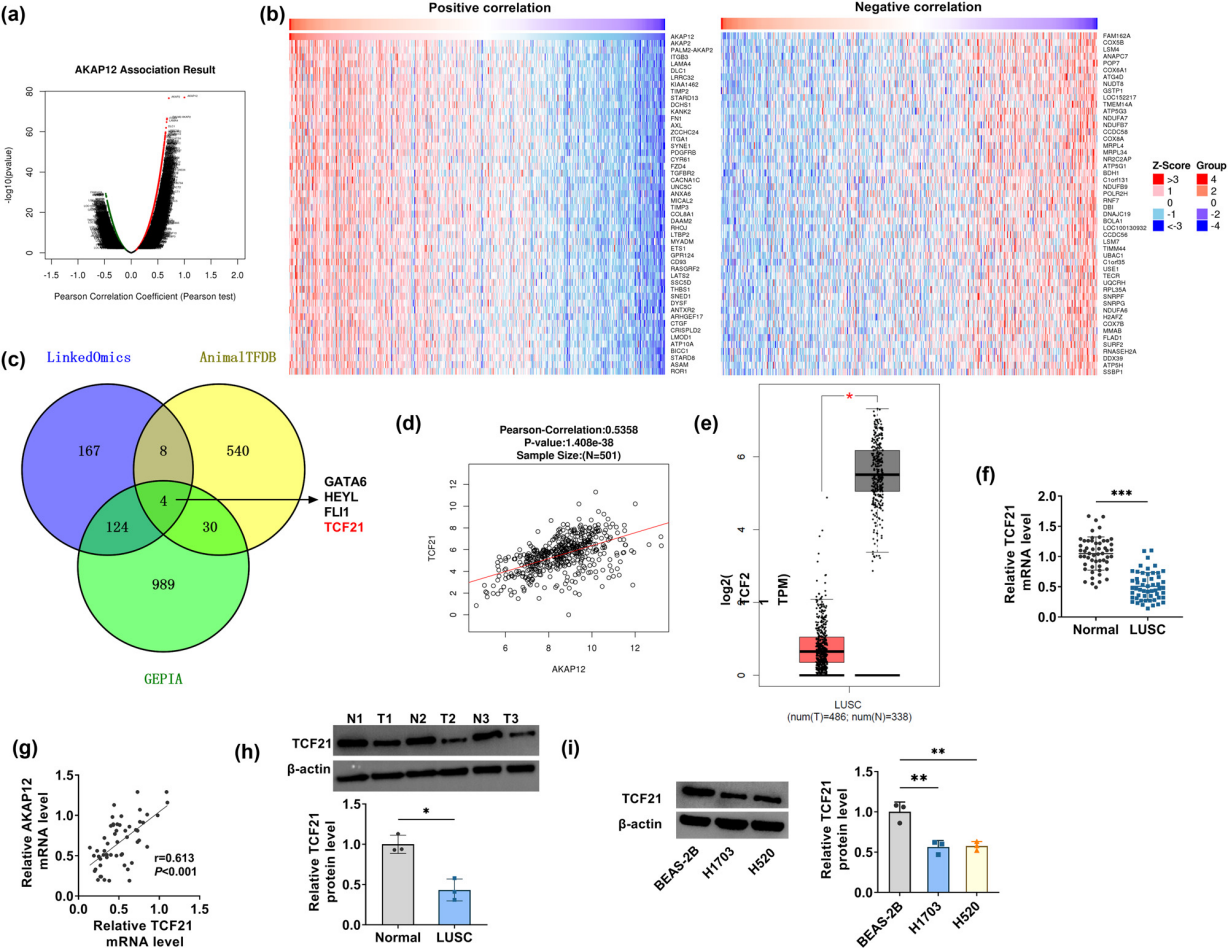
TCF21 expression in LUSC tissues and cells. (a) LinkedOmics database showed the genes associated with AKAP12 expression in LUSC tissues (green-negative, red-positive). (b) TOP50 genes with positive (left) and negative (right) correlations were shown. (c) Venn diagram screened the genes from LinkedOmics database, GEPIA dataset, and AnimalTFDB database. (d) LinkedOmics database showed the correlation between TCF21 and AKAP12 expression in LUSC tissues. (e) GEPIA database revealed TCF21 expression in LUSC tumor tissues and normal tissues. (f) TCF21 mRNA level was measured using qRT-PCR in LUSC tumor tissues (*n* = 54) and adjacent normal tissues (*n* = 54). (g) Pearson correlation coefficient analyzed the correlation between TCF21 and AKAP12 expression in LUSC tissues. (h) TCF21 protein level in LUSC tumor tissues (*n* = 3) and adjacent normal tissues (*n* = 3) was examined by WB. (i) TCF21 protein level was tested using WB in BEAS-2B, H1703, and H520 cells. *P < 0.05, **P < 0.01, ***P < 0.001.

Volcano plot revealed the genes associated with AKAP12 expression in LUSC tissues in the LinkedOmics database (Figure 3a), where TOP50 genes with positive (left) and negative (right) correlations (Pearson correlation >0.5, *P* < 0.05) are shown in Figure 3b. To search for the upstream transcription factors of AKAP12, LinkedOmics database (selected genes positively correlated with AKAP12 expression in LUSC tissues, Pearson correlation >0.5, *P* < 0.05), GEPIA dataset (selected the downregulated genes in LUSC tissues, *P* < 0.05, log2FoldChange <−1) and AnimalTFDB database (selected the transcription factors with binding sites to AKAP12 promoters) were selected, and the intersection was taken to screen out four genes (Figure 3c). TCF21 has been confirmed to play tumor suppressor effect in lung cancer [18], but its mechanism in LUSC is still unclear. Therefore, TCF21 was selected for further exploration. The LinkedOmics database indicated that TCF21 was positively correlated with AKAP12 expression in LUSC tissues (Figure 3d), and the GEPIA database suggested that TCF21 was downregulated in LUSC tissues (Figure 3e). Through qRT-PCR, we confirmed the decreased TCD21 expression in LUSC tissues (Figure 3f), and further analysis confirmed the positive correlation between AKAP12 and TCF21 levels (Figure 3g). The detection of TCF21 protein expression suggested that TCF21 was lowly expressed in LUSC tissues and cells (Figure 3h and i).

### 3.4 TCF21 promoted AKAP12 expression through binding to its promoter region

**Figure 4:**
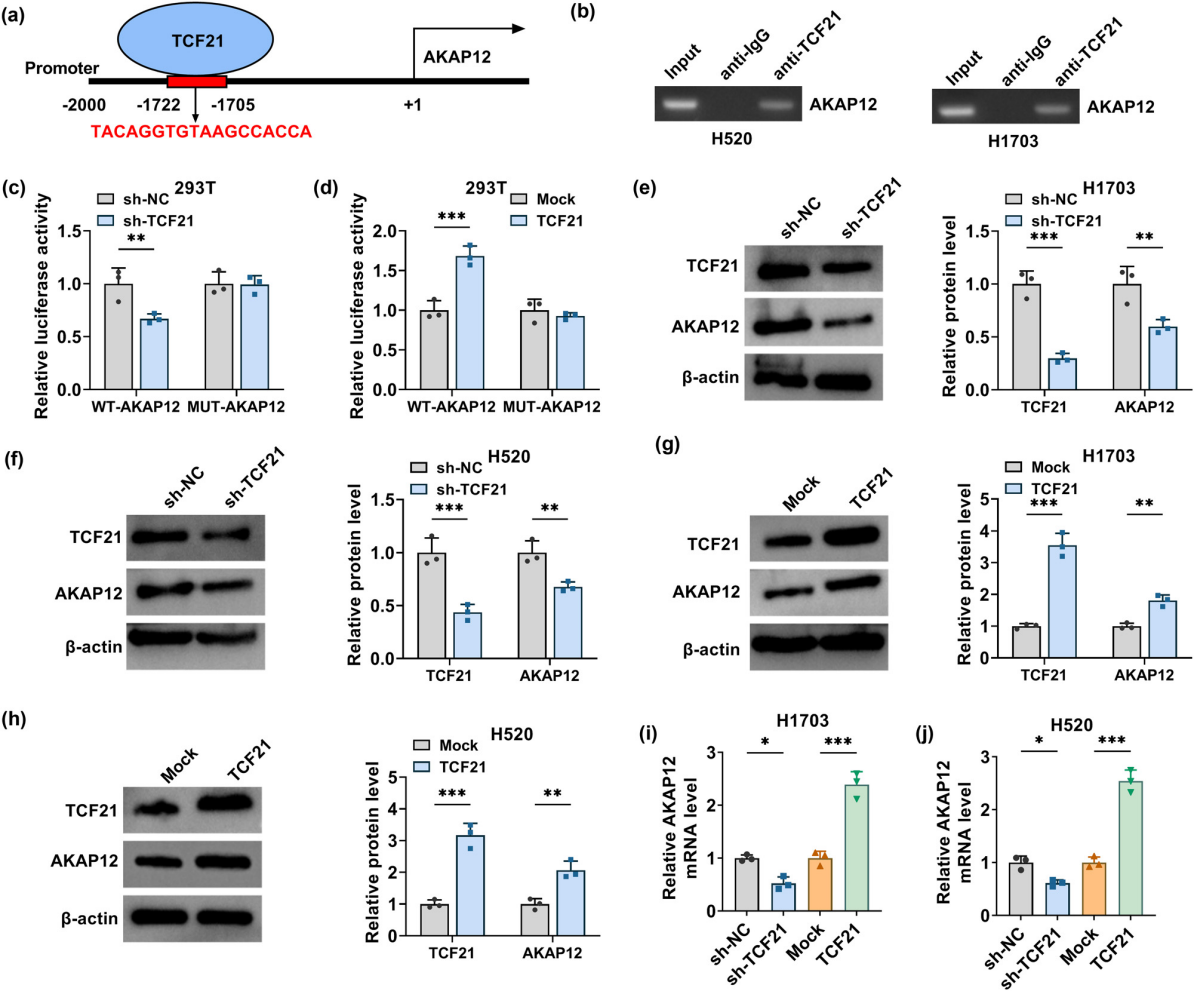
TCF21 interacted with AKAP12. (a) The binding sites of TCF21 in AKAP12 promoter region were shown. ChIP assay (b) and dual-luciferase reporter assay (c and d) were performed to confirm the interaction between TCF21 and AKAP12. (e–h) TCF21 and AKAP12 protein levels were tested by WB in H1703 and H520 cells transfected with sh-NC/sh-TCF21/Mock/TCF21 overexpression vector. (i and j) AKAP12 mRNA level was tested by qRT-PCR in H1703 and H520 cells transfected with sh-NC/sh-TCF21/Mock/TCF21 overexpression vector. *P < 0.05, **P < 0.01, ***P < 0.001.

Animal TFDB database predicted that TCF21 had binding sites in the promoter region of AKAP12 (Figure 4a). Then, a ChIP assay was performed, and we confirmed that immunoprecipitated AKAP12 promoter fragments were significantly enriched in anti-TCF21 (Figure 4b). Moreover, only the luciferase activity of the WT-AKAP12 vector rather than the MUT-AKAP12 vector could be reduced by sh-TCF21 and promoted by TCF21 overexpression (Figure 4c and d). Besides, downregulated TCF21 using sh-TCF21 could significantly decrease AKAP12 protein level (Figure 4e and f), and upregulated TCF21 using TCF21 overexpression vector also markedly increased AKAP12 protein level (Figure 4g and h). Similarly, we obtained the same results at the mRNA level (Figure 4i and j).

### 3.5 TCF21 reduced LUSC cell functions by decreasing AKAP12 expression

**Figure 5:**
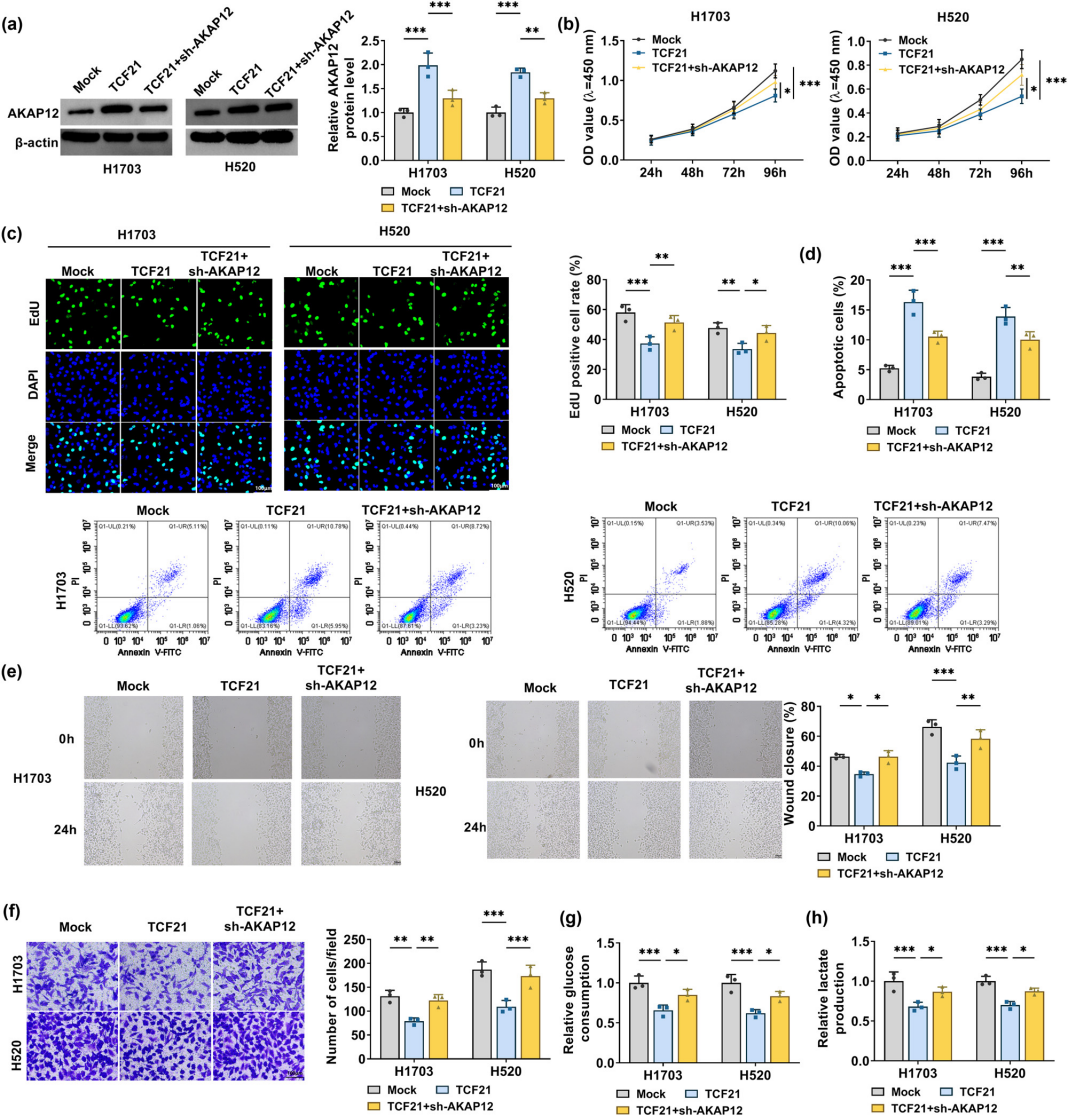
Effects of TCF21 and sh-AKAP12 on LUSC cell functions. H1703 and H520 cells were transfected with Mock, TCF21 overexpression vector, and sh-AKAP12. (a) AKAP12 protein level was tested using WB. Cell proliferation, apoptosis, migration, and invasion were determined using CCK8 assay (b), EdU assay (c), flow cytometry (d), wound healing assay (e), and transwell assay (f). (g and h) Glucose consumption and lactate production were detected to measure cell glycolysis. *P < 0.05, **P < 0.01, ***P < 0.001.

To explore TCF21 roles in LUSC progression and whether TCF21 regulates AKAP12 to mediate the LUSC process, H1703 and H520 cells were transfected with TCF21 overexpression vector and sh-AKAP12. AKAP12 protein expression enhanced by TCF21 overexpression vector could be abolished by sh-AKAP12 (Figure 5a). TCF21 overexpression inhibited LUSC cell viability, decreased EdU positive cell rate, and promoted apoptotic cell rate, while these effects were reversed by AKAP12 knockdown (Figure 5b–d). Elevated TCF21 expression also contributed to the reduction of wound closure rate, invaded cell numbers, glucose consumption and lactate production in H1703 and H520 cells, and AKAP12 knockdown eliminated these effects (Figure 5e–h).

### 3.6 AKAP12 reduced LUSC tumorigenesis *in vivo*

**Figure 6:**
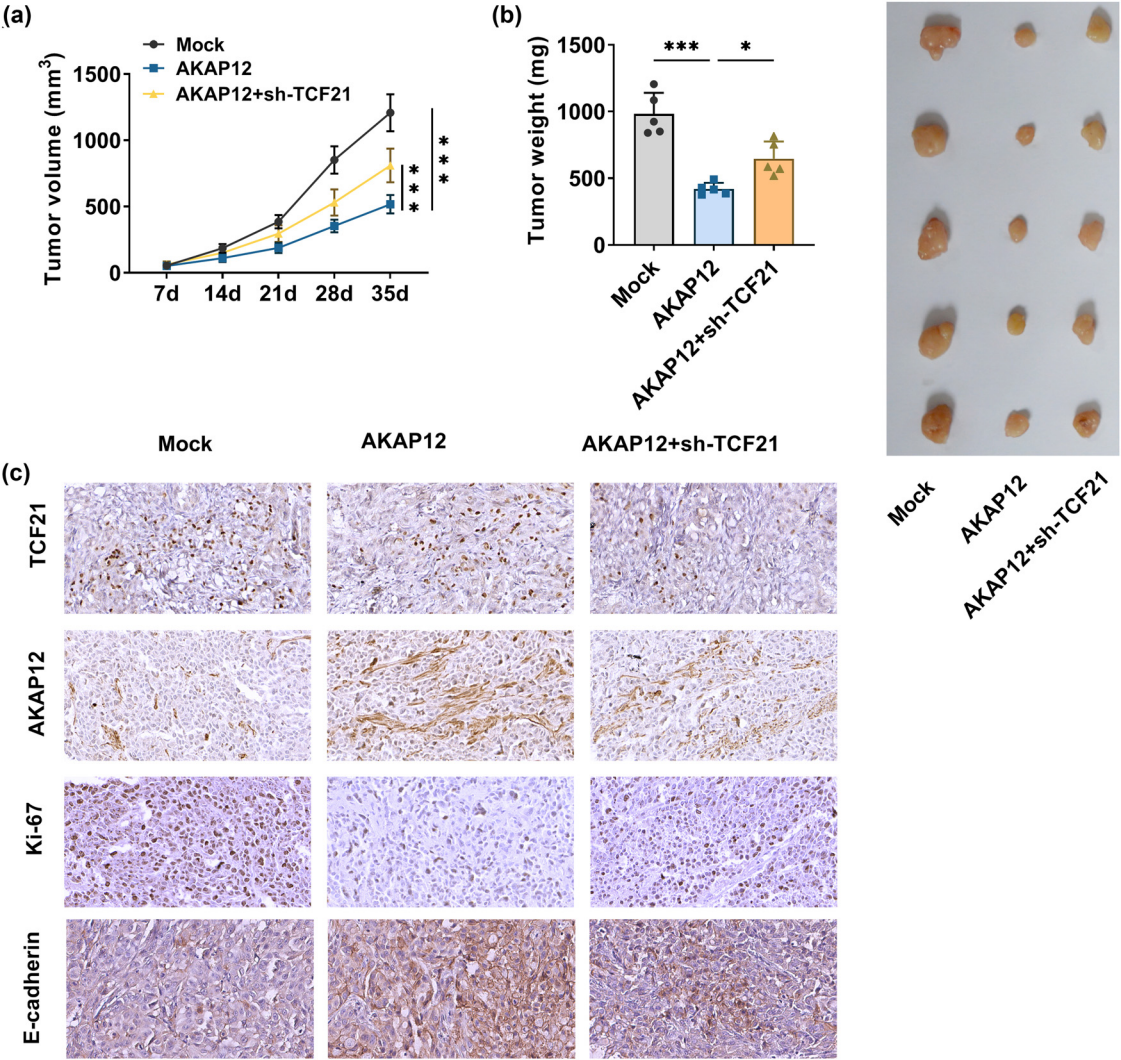
Effects of AKAP12 and sh-TCF21 on LUSC tumorigenesis *in vivo*. (a) Tumor volume was detected in each group every week. (b) Tumor weight was measured in each group. (c) TCF21-, AKAP12-, Ki-67-, and E-cadherin-positive cells in tumor tissues of each group were examined by IHC staining. *P < 0.05, ***P < 0.001.

To further confirm this, we constructed xenograft tumor models. AKAP12 overexpression significantly reduced tumor volume and weight, while this effect was partially abolished by TCF21 knockdown (Figure 6a and b). Through IHC staining, we observed that AKAP12 overexpression enhanced the positive cells of AKAP12 and metastasis marker E-cadherin while decreased the positive cells of proliferation marker Ki-67, without affecting TCF21 positive cells. However, the positive cells of TCF21, AKAP12, and metastasis marker E-cadherin were reduced, while proliferation marker Ki67-positive cells were enhanced in the tumor tissues of the AKAP12 + sh-TCF21 group (Figure 6c). These data confirmed that TCF21-mediated AKAP12 could reduce LUSC tumorigenesis.

## 4 Discussion

LUSC is a pathological type of lung cancer with a very poor prognosis, characterized by rapid progression and aggressive growth [22,23]. Due to the lack of specific driver genes as therapeutic targets, the prognosis of LUSC patients is poor [24]. Therefore, the present study aims to investigate the underlying molecular mechanisms of LUSC to provide an effective target for its treatment.

AKAP12 can regulate key mediators to control oncogenic signaling pathways in a spatiotemporal manner [25]. In many cancers, AKAP12 downregulation is usually associated with malignant tumor progression and metastasis [25]. Reportedly, both AKAP12 mRNA and protein levels were downregulated in patients with gastric adenocarcinoma, which might have good clinical prospects as a prognostic target [26]. AKAP12 has been reported to be involved in regulating LUSC progression and could provide guidance for the clinical treatment of LUSC [14]. However, there are fewer studies on the mechanism of AKAP12 in LUSC. Through database analysis, we clarified the low expression of AKAP12 in LUSC tissues, which was confirmed by further qRT-PCR and WB. Furthermore, we detected that AKAP12 suppressed LUSC cell growth, metastasis, and glycolysis *in vitro*, as well as restrained LUSC tumorigenesis *in vivo*, showing that AKAP12 might be an effective target to inhibit LUSC progression.

Studies have found that TCF21 binds to DNA to mediate cell fate and differentiation [27]. Ni et al. suggested that TCF21 was low-expressed in pancreatitis mice, and its upregulation promoted pancreatic stellate cell proliferation and migration [28]. Besides, TCF21 inhibited melanoma cell proliferation and invasion via miR-10a-5p/LIN28B [17]. Tian et al. revealed that LINC01936 hindered LUSC cell growth and metastasis, which was achieved by regulating TCF21 expression [29]. Previous studies have revealed that promoter methylation of the p16INK4a gene occurs more frequently in NSCLC patients before treatment and in patients with leukopenia, suggesting that promoter methylation of the p16INK4a gene may be associated with late clinical stage [30]. TCF21 DNA methylation levels were higher in hepatocellular carcinoma tissues than in adjacent non-tumor lung tissues [31]. However, the role of TCF21 in LUSC progression remains to be revealed. In this, we found that TCF21 was an upstream transcription factor of AKAP12, and TCF21 expression was related to AKAP21 expression in LUSC tissues. In terms of mechanisms, TCF21 bound the AKAP12 promoter region to facilitate its expression. Meanwhile, we observed that AKAP12 knockdown reversed the reduction effect of TCF21 overexpression on LUSC cell growth, metastasis, and glycolysis. In an animal study, we observed the number of positive cells of proliferation marker Ki-67 and metastasis marker E-cadherin using IHC staining to evaluate the proliferation and metastasis of tumor tissue. The results showed that Ki-67-positive cells were enhanced and E-cadherin-positive cells were reduced in the AKAP12 + sh-TCF21 group, showing that sh-TCF21 reversed AKAP12-mediated LUSC tumorigenesis inhibition. Thus, we believed that TCF21 enhanced AKAP12 expression to repress LUSC cell progression.

Of course, there are some limitations to this study. AKAP12 is a member of the AKAP family and can act as a scaffold protein to be involved in the regulation of several signaling molecules. AKAP12 mitigates liver damage by targeting the PI3K/AKT/PCSK6 pathway, which is one of the main mechanisms of AKAP12 in liver cells [32]. AKAP12 may also affect cell proliferation, migration, and tumor metastasis in LUSC through other pathways, but these mechanisms require further research to clarify in the future.

**Figure 7:**
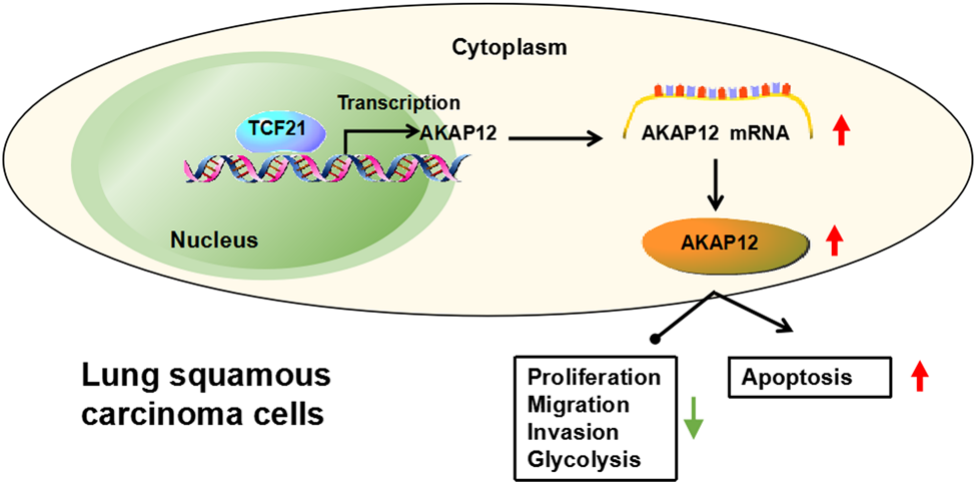
Mechanistic diagram of this study. AKAP12 inhibited LUSC cell proliferation, migration, invasion, glycolysis, and promoted apoptosis, which expression was promoted by TCF21.

Taken together, our study revealed a novel mechanism of AKAP12 as a tumor suppressor in LUSC. These data showed that AKAP12, regulated by TCF21, repressed LUSC malignant progression by inhibiting cell growth, metastasis, and glycolysis (Figure 7). The proposed TCF21/AKAP12 axis might provide a potential target for LUSC treatment. However, the deficiency of this study is that the downstream pathway has not been investigated, which will be further revealed in the future.
